# TERMA Framework for Biomedical Signal Analysis: An Economic-Inspired Approach

**DOI:** 10.3390/bios6040055

**Published:** 2016-11-02

**Authors:** Mohamed Elgendi

**Affiliations:** 1 Department of Obstetrics & Gynecology, University of British Columbia, Vancouver, BC V6Z 2K5, Canada; moe.elgendi@gmail.com; Tel.: +1-604-600-4139; 2 Department of Electrical and Computer Engineering, University of British Columbia, Vancouver, BC V6T 1Z4, Canada

**Keywords:** trend-following, lagging indicator, crossover, quasi-periodic signals, eventogram transform, mobile health, global health, internet-of-things devices, wearable sensors, point-of-care devices

## Abstract

Biomedical signals contain features that represent physiological events, and each of these events has peaks. The analysis of biomedical signals for monitoring or diagnosing diseases requires the detection of these peaks, making event detection a crucial step in biomedical signal processing. Many researchers have difficulty detecting these peaks to investigate, interpret and analyze their corresponding events. To date, there is no generic framework that captures these events in a robust, efficient and consistent manner. A new method referred to for the first time as two event-related moving averages (“TERMA”) involves event-related moving averages and detects events in biomedical signals. The TERMA framework is flexible and universal and consists of six independent LEGO building bricks to achieve high accuracy detection of biomedical events. Results recommend that the window sizes for the two moving averages ($W_{1}$ and $W_{2}$) have to follow the inequality $\left(8\times W_{1}\right)\geq W_{2}\geq \left(2\times W_{1}\right)$. Moreover, TERMA is a simple yet efficient event detector that is suitable for wearable devices, point-of-care devices, fitness trackers and smart watches, compared to more complex machine learning solutions.

## 1. Introduction and Motivation

Clinicians use biomedical signals, such as electrocardiogram (ECG), photoplethysmogram (PPG), acceleration photoplethysmogram (APG) and heart sound signals, to screen and diagnose various cardiac abnormalities. Collecting these biomedical signals is relatively easy and inexpensive when compared to invasive alternatives [1]. Therefore, the analysis of biomedical signals has been extensively investigated over the past two decades. Many algorithms using a variety of mathematical formulae have been published to analyze biomedical signals; however, there is no generic methodology with a clear framework that can be used to analyze these signals. Such a generic methodology may provide physicians with greater insights about a patient’s health through non-invasive measures.

A generic framework that has been well established in the field of economics to analyze financial data is the use of two moving averages. A moving average is commonly used with time series data to smooth out short-term fluctuations and highlight longer term trends or cycles. The use of one moving average is a common analysis tool used by traders to identify trend directions.

**Figure 1:**
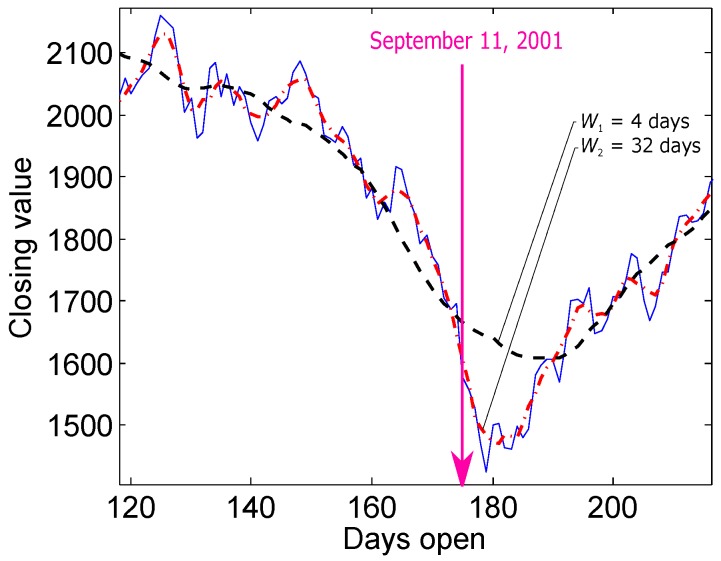
Filtered and unfiltered closing values of the NASDAQ composite index for calendar year 2001. The dashed black line is the first moving average with a four-day window length, and the dotted red line is the second moving average with a 32-day window length.

Two moving averages have been used together to generate crossover signs [2,3]. A crossover occurs when a faster (shorter) moving average crosses a slower (longer) moving average [2,3], and these crossovers are considered as buy and sell indicators. The use of two moving averages succeeds in detecting the critical events in trading. Looking at the NASDAQ composite index for calendar year 2001, if the closing values are filtered, much of the day-to-day market variations can be removed. For example, with the use of two moving averages—the shorter with a four-day window length and the longer with a 32-day window length—the amount of the remaining variations can be controlled. The moving average with the longer window length works as a threshold to the moving average with the shorter window length and, consequently, presents a crossover as an indicator of a critical event, as shown in Figure 1.

It is common practice in biomedical signal analysis to use the moving average as a filter. It is important to note that the moving average step has not been previously used in the decision making. For example, the moving average used in [4,5,6] was only a filtering step, and it was not used for decision making (thresholding) as it is applied in economics. The implementation of the moving average can be highly numerically efficient (simple, fast, and with fewer calculations required). Therefore, the idea of using two moving averages is promising for analyzing biomedical signals.

Analyzing real-time biomedical signals collected by a battery-driven device needs to be fast and feasible in real time, despite the existing limitations in terms of memory and processor capability. The same holds for the ability to analyze large biomedical signals collected over one or more days. The main goal of this study is to produce a methodology that can be used for detecting different types of events in different types of biomedical signals using two event-related moving averages (TERMA). The window sizes of the moving averages depend on prior knowledge of the expected duration of the event to be detected. In this paper, I will demonstrate and discuss how TERMA can be used to detect events in different research areas related to biomedical signals.

## 2. Methods

### 2.1. Data Used

In this section, four different biomedical signals are used: ECG, PPG, APG, and heart sounds signals. Each biomedical signal has it is own unique set of features and events. A single ECG heartbeat signal consists of P, QRS, and T waves, a single PPG pulse signal consists of a systolic wave, a single APG heartbeat signal consists of *a*, *b*, *c*, *d*, and *e* waves, and a single heart beat signal consists of two waves: S1 and S2. The databases used to detect these events are:

- For QRS detection in ECG signals: Eleven ECG databases are used to evaluate the robustness of the TERMA-based QRS detection algorithm. The 11 representative datasets are published on PhysioNet (https://physionet.org/) and represent different subject groups and recording conditions, such as sampling rates (between 128 Hz and 1 kHz) and interferences. Following is a brief description of the 11 datasets: the MIT-BIH Arrhythmia Database with 109,984 beats [7], the QT Database with 111,301 beats [8], the T Wave Alternans Database with 19,003 beats, selected for its wide range of pathological conditions [9], the Intracardiac Atrial Fibrillation Database with 6705 beats [10], the ST Change Database with 76,181 beats featuring stress ECGs [11], the Supraventricular Arrhythmia Database with 184,744 beats [12], the Atrial Fibrillation Termination Database with 7618 beats [13], the Fantasia Database with 278,996 beats from relaxed healthy subjects [14], the Noise Stress Test Database with 26,370 beats recorded under noise conditions typical of clinical environments [15], the St. Petersburg Institute of Cardiological Technics Arrhythmia Database with 175,918 beats [16] and the Normal Sinus Rhythm Database with 183,092 beats [16]. In the Fantasia Database, one record (‘f2y02’) was corrupted and was therefore excluded. Lead I of every record in these datasets was used without any exclusion. The R peaks in all of these publicly-available datasets were annotated. The training set was the MIT-BIH Arrhythmia Database, while the test set consisted of the other 10 databases.
- For T wave detection in ECG signals: Two annotated databases were used, the MIT-BIH Database [7] and the QT Database [8]. The latter was the training set, and the former was the test set.
- For systolic wave detection in PPG signals: One annotated Heat-Stress PPG Database [17] consists of 5071 beats of 40 healthy, heat-acclimatized emergency responders (30 males and 10 females). The PPG data were collected at a sampling rate of 367 Hz, and the duration of each recording was 20 s. The data used in the training set were the PPG signals measured at rest, while the data used in the test set were the PPG signals measured after three simulated heat stress exercises.
- For *a*, *b*, *c*, *d* and *e* waves detection in APG signals: One annotated Heat-Stress PPG Database [18] consists of 1469 beats of 27 healthy volunteers (males). The PPG data were collected at a sampling rate of 200 Hz, and the duration of each recording was 20 s. The data used in the training set were the APG signals after 1 h of exercise, while the data used in the test set consisted of the APG signals measured at rest and after 2 h of exercise.
- For S1 and S2 detection in heart sounds: One annotated Heart Sounds Database [19] was used that contains the heart sounds of 22 subjects with and without pulmonary artery hypertension (PAH). The heart sounds were recorded using a 3 M Littmann 3200 digital stethoscope over 20 s with sampling frequencies of 4000 Hz. Heart sounds were recorded sequentially at the second left intercostal space and the cardiac apex for 20 s. The data used in the training set were that of 11 subjects with mean pulmonary arterial pressure (PAP) ≥25 mmHg collected from the apex site, while the data used in the test set were that of 11 subjects with mean PAP <25 mmHg collected from the apex site, 11 subjects with mean PAP ≥25 mmHg collected from the second left intercostal space (2 L) site and 11 subjects with mean PAP <25 mmHg collected from the 2 L site.

### 2.2. TERMA Framework

**Figure 2:**
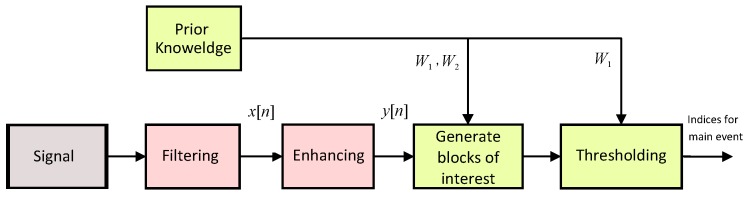
Flowchart of the two event-related moving averages (TERMA) algorithm for detecting the main event in a quasi-periodic signal. The algorithm consists of six LEGO building bricks: signal, filtering, enhancing, generating blocks of interest, thresholding and prior knowledge.

In this section, a new, knowledge-based, numerically-efficient and robust method is proposed to detect main events in biomedical signals using the TERMA algorithm. The structure of the TERMA algorithm is shown in Figure 2.

**Figure 3:**
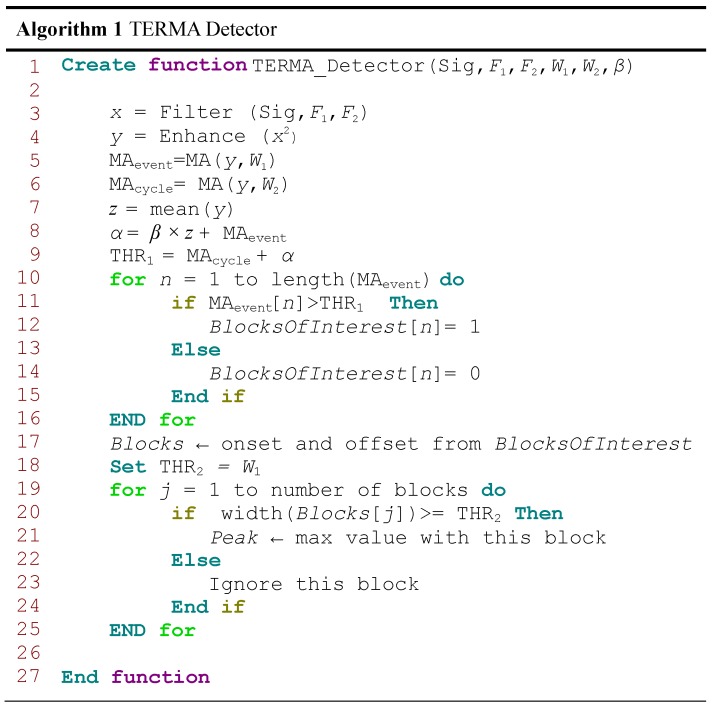
Pseudocode for the TERMA-based detector function. The function has six inputs: biomedical signal (Sig), $F_{1}$, $F_{2}$, $W_{1}$, $W_{2}$ and *β*. The band-pass filter will be determined by the frequency band $F_{1}$–$F_{2}$ Hz, while $W_{1}$ and $W_{2}$ are the window sizes of the two moving averages ${\mathrm{MA}}_{\mathrm{event}}$ and ${\mathrm{MA}}_{\mathrm{cycle}}$, respectively. However, *β* is used to calculate the statistical threshold *α*.

It is clear that prior knowledge of TERMA parameters supports the decision making in both stages, generating blocks of interest and thresholding. The more precise the prior knowledge is, the higher the overall performance and detection accuracy. The pseudocode of the TERMA detector is shown in Figure 3.

#### 2.2.1. Prior Knowledge

TERMA prior knowledge about the duration of the main events of the biomedical signals can assist in feature extraction and, thus, support the decision making of the algorithm. Four parameters are required as prior knowledge: the frequency band ($F_{1}$–$F_{2}$), event-related durations $W_{1}$ and $W_{2}$ and the offset fraction (*β*). Usually, the TERMA prior knowledge for these parameters is not reported in the literature.

In the literature, this needed prior knowledge (TERMA parameter values) of all biological events has not been reported yet. Therefore, it is recommended to take a subset of the data to determine the duration of the main events via an optimization step. In other words, the output of the optimization step will be used as prior knowledge for the rest of the dataset.

The optimization step has five decision variables: $F_{1}$, $F_{2}$, $W_{1}$, $W_{2}$ and *β*. The improvement in one objective will result in the worsening of at least one other objective, generating Pareto solutions [20]. Any change in these parameters affects the overall performance of the proposed algorithm. The five decision variables are interrelated and cannot be optimized in isolation. Our goal is to find the Pareto optimal point, within all possible Pareto solutions, for this multi-objective problem. An aggregate objective function denoted by *J* to combine two objective functions into a scalar function is defined as follows:

$$\begin{array}{cc} & \max_{F_{1},F_{2},W_{1},W_{2},\beta }\;\;J=\frac{1}{2}\left\{\left[\mathrm{SE}\left(F_{1},F_{2},W_{1},W_{2},\beta \right)\right]+\left[+P\left(F_{1},F_{2},W_{1},W_{2},\beta \right)\right]\right\}\\ \mathrm{subject}\;\mathrm{to} & \\ & f_{1min}\leq F_{1}\leq {f_{1}}_{max}\;,\\ & f_{2min}\leq F_{2}\leq {f_{2}}_{max}\;,\\ & w_{1min}\leq W_{1}\leq {w_{1}}_{max}\;,\\ & w_{2min}\leq W_{2}\leq {w_{2}}_{max}\;,\\ & b_{min}\leq \beta \leq b_{max}\;, \end{array}$$

where *J* is the overall accuracy, which is defined as the average of sensitivity (SE) and positive predictivity (+P). SE and +P are the two objective functions to be maximized jointly. The Pareto frontier is formed with solutions (the values of five decision variables) which optimise them both. Finding the optimal Pareto point goes through a brute-force search over all parameters, which is time consuming, but once achieved, the optimal solution will be used as is for the implementation.

#### 2.2.2. Band-Pass Filter

Morphologies of normal and abnormal events in biosignals differ widely. Biosignals are often corrupted by noise from many sources; therefore, band-pass filtering is an essential first step for nearly all event detection algorithms. The purpose of band-pass filtering is to remove the baseline wander and high frequencies that do not contribute to detecting these events. A band-pass filter is typically used as a bidirectional Butterworth implementation [21]. It offers good transition band characteristics at low coefficient orders, which makes it efficient to implement [21]. All research was carried out using the TERMA method, a third-order Butterworth filter with a passband of $F_{1}$–$F_{2}$ Hz to remove baseline wander and high frequencies [17,19,22,23,24,25], where $F_{1}$ is the starting frequency and $F_{2}$ is the stopping frequency, as shown in Line 3 in Figure 3.

#### 2.2.3. Enhancing

The signal is squared point by point to enhance large values and boost high-frequency components using the following equation:

(1) $$y\left[n\right]={\left(x\left[n\right]\right)}^{2}$$

as shown in Line 4 in Figure 3.

#### 2.2.4. Generating Blocks of Interest

Blocks of interest are generated using two event-related moving averages. The first moving average ${\mathrm{MA}}_{\mathrm{event}}$ is used to extract a specific event (within a cycle), while the second moving average ${\mathrm{MA}}_{\mathrm{cycle}}$ extracts the cycle (regularly-repeating events). Next, an event-related threshold is applied to the generated blocks to distinguish the blocks that contain the event peaks from the blocks that include noise. The purpose of the event moving average (${\mathrm{MA}}_{\mathrm{event}}$) is to smooth out multiple peaks corresponding to the event length to emphasize and extract the event area:

(2) $$\begin{array}{c} {\mathrm{MA}}_{\mathrm{event}}\left[n\right]=\frac{1}{W_{1}}(y\left[n-\left(W_{1}-1\right)/2\right]+\cdots \\ +y\left[n\right]+\cdots +y[n+\left(W_{1}-1\right)/2]) \end{array}$$

where $W_{1}$ is the approximate duration of a specific event, rounded to the nearest odd integer, and *n* is the number of data points. The value of $W_{1}$ is determined based on the prior knowledge discussed above.

The purpose of the cycle moving average (${\mathrm{MA}}_{\mathrm{cycle}}$) is similar to that of the ${\mathrm{MA}}_{\mathrm{event}}$, but emphasizes the cycle area that contains the event of interest to be used as a threshold for the first moving average (${\mathrm{MA}}_{\mathrm{event}}$):

(3) $$\begin{array}{c} {\mathrm{MA}}_{\mathrm{cycle}}\left[n\right]=\frac{1}{W_{2}}(y\left[n-\left(W_{2}-1\right)/2\right]+\cdots \\ +y\left[n\right]+\cdots +y[n+\left(W_{2}-1\right)/2]) \end{array}$$

where $W_{2}$ is the approximate duration of a cycle (or heartbeat), rounded to the nearest odd integer, and *n* is the number of data points. The value of $W_{1}$ is determined based on the prior knowledge discussed above.

**Figure 4:**
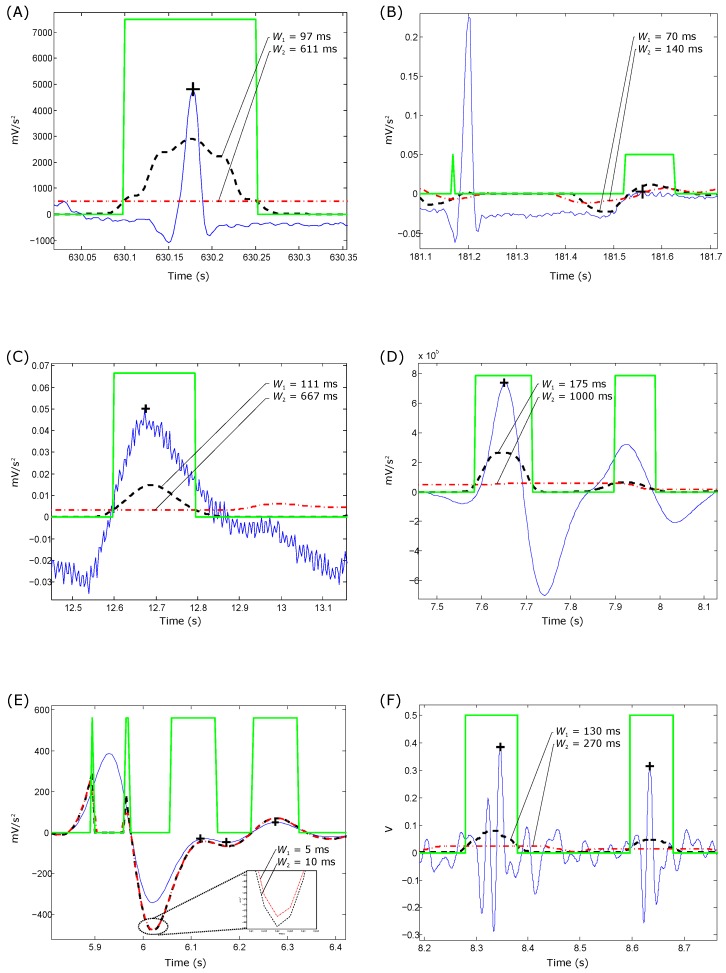
Demonstrating the effectiveness of TERMA in detecting events in biomedical signals. (**A**) QRS detection; (**B**) T wave detection; (**C**) systolic wave detection; (**D**) *a* wave detection; (**E**) c,d, and *e* wave detection; and (**F**) first and second heart sounds detection. The dashed black line is the ${\mathrm{MA}}_{\mathrm{event}}$ with $W_{1}$, and the dotted red line is the ${\mathrm{MA}}_{\mathrm{cycle}}$ with $W_{2}$. The peak of the investigated event is detected using TERMA (represented by a black plus sign) within the blocks of interest (represented by a green square pulse).

The blocks of interest are generated based on the two moving averages discussed. In other words, applying the second moving average ${\mathrm{MA}}_{\mathrm{cycle}}$ as a threshold to the first moving average ${\mathrm{MA}}_{\mathrm{event}}$ produces blocks of interest, as shown in Figure 4. However, the use of ${\mathrm{MA}}_{\mathrm{cycle}}$ without an added offset reduces the detection accuracy because of its sensitivity to a low signal-to-noise ratio (SNR).

Here, the SNR is defined as the ratio of the mean signal of a region of interest to its standard deviation [26], which means if the statistical mean of the signal increases, the SNR increases. This leads to introducing an offset based on the statistical mean of the signal as:

(4) $$\alpha =\beta \times \bar{z}$$

where *β* is the fraction of the $\bar{z}$ signal that needs to be removed, $\bar{z}$ is the statistical mean of the squared ECG signal *z* and *α* is an offset for the threshold ${\mathrm{MA}}_{\mathrm{cycle}}$ signal. Therefore, *α* refers to the offset, while *β* refers to the offset fraction.

In short, to increase the accuracy of detecting events in noisy biosignals, the dynamic threshold value ${\mathrm{THR}}_{1}$ is calculated by offsetting the ${\mathrm{MA}}_{\mathrm{cycle}}$ signal with *α*, as follows:

(5) $${\mathrm{THR}}_{1}={\mathrm{MA}}_{\mathrm{cycle}}\left[n\right]+\alpha$$

The blocks of interest are then generated by comparing the ${\mathrm{MA}}_{\mathrm{event}}$ signal with ${\mathrm{THR}}_{1}$. If a block is higher than ${\mathrm{THR}}_{1}$, it is classified as a block of interest containing biosignal features (different events) and noise, as shown in Lines 10–16 in Figure 3. By this stage, the blocks of interest are generated and stored in Blocks[n] as a square pulse (ones and zeros). Therefore, the next step is to reject the blocks that result from noise. The rejection should be related to the anticipated block width.

#### 2.2.5. Thresholding

Here, blocks containing undesired (or out of interest) events and noise are rejected using the new ${\mathrm{THR}}_{2}$ threshold. By applying the ${\mathrm{THR}}_{2}$ threshold, the accepted blocks contain only the required events:

(6) $${\mathrm{THR}}_{2}=W_{1}$$

As discussed, the threshold ${\mathrm{THR}}_{2}$ equals $W_{1}$, which corresponds to the anticipated event width. If the block width equals the window size $W_{1}$, then the block contains an event. However, the event duration varies in terms of the durations of the abnormal events within the processed signal. Therefore, the condition is set to capture both normal and abnormal event durations. Therefore, if a block width is greater than or equal to $W_{1}$, it is classified as an event. If not, the block is classified as an undesired event or noise.

#### 2.2.6. Detecting Event Peak

The last stage is finding the maximum absolute value within each block or the event peak.

## 3. Results

The event detection algorithm is typically run using two statistical measures: SE and +P, where SE=TP/(TP+FN) and +P=TP/(TP+FP). Here, TP is the number of true positives (events detected as events), FN is the number of false negatives (events that have not been detected as events) and FP is the number of false positives (non-events detected as events). The SE reports the percentage of true events that were correctly detected by the algorithm. The +P reports the percentage of event detections that were true events.

### 3.1. Training Results

The training dataset for each detection problem is discussed in the Data Used subsection. A rigorous optimization using a brute-force search over all parameters is conducted as follows.

- For QRS detection in ECG signals: The optimization of the beat detector’s spectral window for lower frequency varied from ${f_{1}}_{min}={f_{2}}_{min}=1$ Hz to ${f_{1}}_{max}=10$ Hz, with the higher frequency up to ${f_{2}}_{max}=26$ Hz. All combinations of the frequency band were 1–26 Hz. The window size of the $W_{1}$ ranged from ${w_{1}}_{min}=55$ ms to ${w_{1}}_{max}=111$ ms, whereas the window size of $W_{2}$ changed from ${w_{2}}_{min}=555$ ms to ${w_{2}}_{max}=694$ ms. However, the offset *β* was tested over the range $b_{min}=0\%$ to $b_{max}=10\%$.
- For T wave detection in ECG signals: All combinations of the frequency band ranged from ${f_{1}}_{min}={f_{2}}_{min}=0$ Hz to ${f_{1}}_{max}={f_{2}}_{max}=10$ Hz. The window size of the $W_{1}$ ranged from ${w_{1}}_{min}=40$ ms to ${w_{1}}_{max}=80$ ms, whereas the window size of $W_{2}$ changed from ${w_{2}}_{min}=100$ ms to ${w_{2}}_{max}=200$ ms. However, the offset *β* was tested over the range $b_{min}=0\%$ to $b_{max}=10\%$.
- For systolic wave detection in PPG signals: The lower frequency resulted in a value from $f_{1min}=0.5$ Hz to ${f_{1}}_{max}=1$ Hz, while the higher frequency resulted in a value from $f_{2min}=7$ Hz to ${f_{2}}_{max}=15$ Hz. The window size of $W_{1}$ varied from ${w_{1}}_{min}=54$ ms to ${w_{1}}_{max}=111$ ms, whereas the window size of $W_{2}$ varied from ${w_{2}}_{min}=545$ ms to ${w_{2}}_{max}=694$ ms. The offset *β* was tested over the range $b_{min}=0\%$ to $b_{max}=10\%$.
- For *a* and *b* wave detection in APG signals: The lower frequency resulted in a value from $f_{1min}=0.5$ Hz to ${f_{1}}_{max}=1$ Hz, while the higher frequency resulted in a value from $f_{2min}=7$ Hz to ${f_{2}}_{max}=15$ Hz. The window size of $W_{1}$ varied from ${w_{1}}_{min}=100$ ms to ${w_{1}}_{max}=200$ ms, whereas the window size of $W_{2}$ varied from ${w_{2}}_{min}=1000$ ms to ${w_{2}}_{max}=1250$ ms. The offset *β* was tested over the range $b_{min}=0\%$ to $b_{max}=10\%$.
- For *c*, *d* and *e* wave detection in APG signals: The lower frequency varied from ${f_{1}}_{min}=0.5$ Hz, while the higher frequency varied from $f_{2min}=4$ Hz to ${f_{2}}_{max}=10$ Hz. The window size of $W_{1}$ varied from ${w_{1}}_{min}=5$ ms to ${w_{1}}_{max}=25$ ms, whereas the window size of $W_{2}$ varied from ${w_{2}}_{min}=10$ ms to ${w_{2}}_{max}=15$ ms, while the range of *β* varied from $b_{min}=0\%$ to $b_{max}=10\%$.
- For S1 and S2 detection in heart sounds: The frequency band was optimized over from $f_{1min}={f_{2}}_{min}=0$ Hz to ${f_{1}}_{max}={f_{2}}_{max}=2000$ Hz; $W_{1}$ varied from ${w_{1}}_{min}=20$ ms to ${w_{1}}_{max}=200$ ms; $W_{2}$ varied from ${w_{2}}_{min}=30$ ms to ${w_{2}}_{max}=400$ ms; and *β* varied from $b_{min}=0\%$ to $b_{max}=10\%$.

The databases used in the optimization process contains abnormal rhythms, different event morphologies, heat stress signals and low SNR signals. Several publications have listed the use of all files in the database, excluding the paced patients, segments and certain beats [27]. However, in the optimization process, all records were used without excluding any segment or beat.

As we have multiple objectives, plotting the Pareto frontier (the objective space of possible Pareto solutions) cannot be achieved. Therefore, all Pareto solutions were sorted in descending order according to the overall accuracy (objective function *J*) [17,19,22,23,24,25]; and thus, the first combination is considered the optimal Pareto solution.

After applying the multi-objective optimization step, the optimal Pareto solution for QRS detection in ECG signals was $F_{1}=8$ Hz, $F_{2}=20$ Hz, $W_{1}=97$ ms, $W_{2}=611$ ms and β=8%, while for T wave detection in ECG signals, the optimal solution was $F_{1}=0.5$ Hz, $F_{2}=10$ Hz, $W_{1}=70$ ms, $W_{2}=140$ ms and β=0%. To detect the systolic waves in PPG signals, the optimal solution was found to be $F_{1}=0.5$ Hz, $F_{2}=8$ Hz, $W_{1}=111$ ms, $W_{2}=667$ ms and β=2%. Moreover, the optimal solution for detecting *a* and *b* waves was $F_{1}=0.5$ Hz, $F_{2}=15$ Hz, $W_{1}=175$ ms, $W_{2}=1000$ ms and β=0%, while the optimal solution for detecting *c*, *d* and *e* waves was found to be $F_{1}=0.5$ Hz, $F_{2}=7$ Hz, $W_{1}=5$ ms, $W_{2}=15$ ms and β=0%. For detecting S1 and S2 in heart sounds, the optimal solution was found to be $F_{1}=0$ Hz, $F_{2}=60$ Hz, $W_{1}=130$ ms, $W_{2}=270$ ms and β=3%.

### 3.2. Testing Results

An optimal event detector is obtained from the training phase. We can then test each detector on its testing dataset ‘straight out of the box’ without any tuning. In other words, the algorithm’s parameters ($F_{1}$, $F_{2}$, $W_{1}$, $W_{2}$ and *β*) do not need to be trained in a real-world application for every subject. The parameters are optimized on a large training set; thus, the robustness of the algorithm can be examined against different databases with different sampling frequencies, and the biosignals can be collected by different doctors in dissimilar conditions.

The performance of the TERMA-based detection algorithm on the testing datasets can be summarized as follows.

- For QRS detection in ECG signals: Interestingly, the TERMA-based QRS detector obtained an SE of 99.29% and a +P of 98.11% over the first lead of the validation databases (10 databases with a total of 1,179,812 beats). When applied to the well-known MIT-BIH Arrhythmia Database, an SE of 99.78% and a +P of 99.87% were attained [22]. The TERMA-based QRS detector outperformed most of the well-known QRS detector, such as Pan–Tompkins [4] (SE of 90.95% and +P of 99.56%) and Hamilton–Tompkins [28] (SE of 99.69% and +P of 99.77%).
- For T wave detection in ECG signals: Over the MIT-BIH Arrhythmia Database, the TERMA-based T wave detector achieved an SE of 99.86% and a +P of 99.65%, which are promising results for handling the non-stationary effects, low SNR, normal sinus rhythm (NSR), left bundle branch block (LBBB), right bundle branch block (RBBB), premature ventricular contraction (PVC) and premature atrial contraction (PAC) in ECG signals [25]. The TERMA-based T wave detector was not compared to other algorithms as the annotation of T-waves was published in 2015. However, the results are very promising, as the scored accuracy over arrhythmic ECG signals is >99.6%.
- For systolic wave detection in PPG signals: The TERMA-based systolic wave detection algorithm was evaluated using 40 records after three heat stress simulations containing 5071 heartbeats, with an overall SE of 99.89% and +P of 99.84% [17]. The TERMA-based systolic detector slightly outperformed existing algorithms, such as Billauer’s [29] (SE of 99.88% and +P of 98.69%), Li’s [30] (SE of 97.9% and +P of 99.93%) and Zong’s [31] (SE of 99.69% and +P of 99.71%).
- For *a* and *b* wave detection in APG signals: The TERMA-based *a* wave detection algorithm demonstrated an overall SE of 99.78% and a +P of 100% over signals that suffer from: (1) non-stationary effects; (2) irregular heartbeats; and (3) low amplitude waves. In addition, the *b* detection algorithm (based on the detection of *a* waves) achieved an overall SE of 99.78% and +P of 99.95% [24]. The TERMA-based *a* and *b* waves detector was not compared to other algorithms, as it is a new area of investigation and is considered a pioneering concept in the field of PPG signal analysis. However, the results are very promising as the scored accuracy over heat-stressed PPG signals is >98%.
- For *c*, *d* and *e* wave detection in APG signals: The performance of the TERMA-based *c*, *d* and *e* wave detector was tested on 27 PPG records collected during rest and after 2 h of exercise, resulting in 97.39% SE and 99.82% +P [23]. The TERMA-based *c*, *d* and *e* waves detector was not compared to other algorithms, as it is a new area of investigation, and the work is a pioneering concept in the field of PPG signal analysis. However, the results are very promising, as the scored accuracy over heat-stressed PPG signals is >97%.
- For S1 and S2 detection in heart sounds: The SE and +P of the TERMA-based S1 and S2 detectors were 70% and 68%, respectively, for heart sounds collected from children with PAH [19]. The TERMA-based heart sounds detector outperformed existing algorithms, such as Liang’s [32] (SE of 59% and +P of 42%), Kumar’s [33] (SE of 19% and +P of 12%), Wang’s [34] (SE of 50% and +P of 45%) and Zhong [35] (SE of 43% and +P of 53%).

Given the simplicity and that the memory and CPU power are not a huge concern nowadays, the proposed TERMA-based algorithm presents a clear advantage over the previously-reported algorithms in terms of detection performance over large datasets and different application problems.

## 4. Discussion

Application of the TERMA-based detectors has been demonstrated in the above section. It is now necessary to further elaborate on the implementation of TERMA-based detectors. It is worth noting that TERMA is simple and clearly laid out in comparison to other detectors published in the literature. For example, well-known algorithms demand more implementation steps [27] and resampling of the biosignals before processing; for example, the Pan–Tompkins algorithm [4] requires a resampling step for any ECG signal not sampled at 200 Hz. Its filters are designed for 200 Hz, so performance will be degraded at other sampling frequencies.

Furthermore, TERMA-based detectors are amplitude-independent, while well-known detectors, such as the Pan–Tompkins algorithm, are amplitude-dependent. Moreover, TERMA-based detectors use an efficient dynamic thresholding, while algorithms, such as the Pan–Tompkins algorithm, have a complicated thresholding step to adjust the threshold. The TERMA-based algorithm does not need to change its threshold based on previous segments.

### 4.1. Frequency Band Choice

In the literature, most of the researchers developed detection algorithms and determined the frequency bands experimentally without justifying their choice. For example, researchers used 5–15 Hz [36], 5–11 Hz [4,5], 4–13.5 Hz [37], 4.1–33.1 Hz [38], 9–30 Hz [39] and 2.2–33.3 Hz [40] as the optimal frequency band to detect QRS complexes in ECG signals. However, the proposed TERMA method extracts the optimal frequency band during the training stage through a rigorous brute force optimization, which is 8–20 Hz in this case, as discussed above.

The choice of frequency band plays a major role in reducing the amount of noise in the processed signals. However, determining a reasonable estimate for the frequency band can be easily carried out on a part of the sample size using the power spectrum of the investigated event [4], which is a relatively easier step compared to determining the window sizes in the TERMA method.

The band-pass filter consists of two filters, the low-pass filter and the high-pass filter. The low-pass filter is used to remove high frequency noise, and the high-pass filter is used to remove low frequency noise. Usually, a Butterworth filter is used due to its simplicity and is characterized by a magnitude response that is maximally flat in the passband and is monotonic overall. MATLAB provides low-pass and high-pass filters with the simple command $butter(m,f{,}^{\prime }low^{\prime })$ and $butter(m,f{,}^{\prime }high^{\prime })$, respectively, where *m* is the filter order and *f* is the normalized cut-off frequency. The purpose of this step is to retain the characteristics of the main events within the processed signal, remove the undesired noise and make the main events more salient.

### 4.2. Window Size Choice

After the noise removal achieved in the previous step, the window sizes of TERMA need to be determined. The two window sizes reflect the event duration and the event repetition period (cyclic duration), which is an individual characteristic that depends on the heart rate and abnormalities and, thus, is hard to predict. It is common that researchers determine the window size of a moving average without a proper justification or reasoning; for example, Pan and Tompkins [4] used one moving average to demarcate the QRS complexes in ECG signals with a window size of 150 ms. However, the proposed TERMA method overcomes the unjustified window sizes and offers two event-related window sizes for the two moving averages. Therefore, the TERMA window sizes depend on the expected duration of the investigated event and repetition period of this event. These window sizes can be adjusted via a predefined dataset or can be optimized over a representative sample during the training phase, as discussed above.

**Figure 5:**
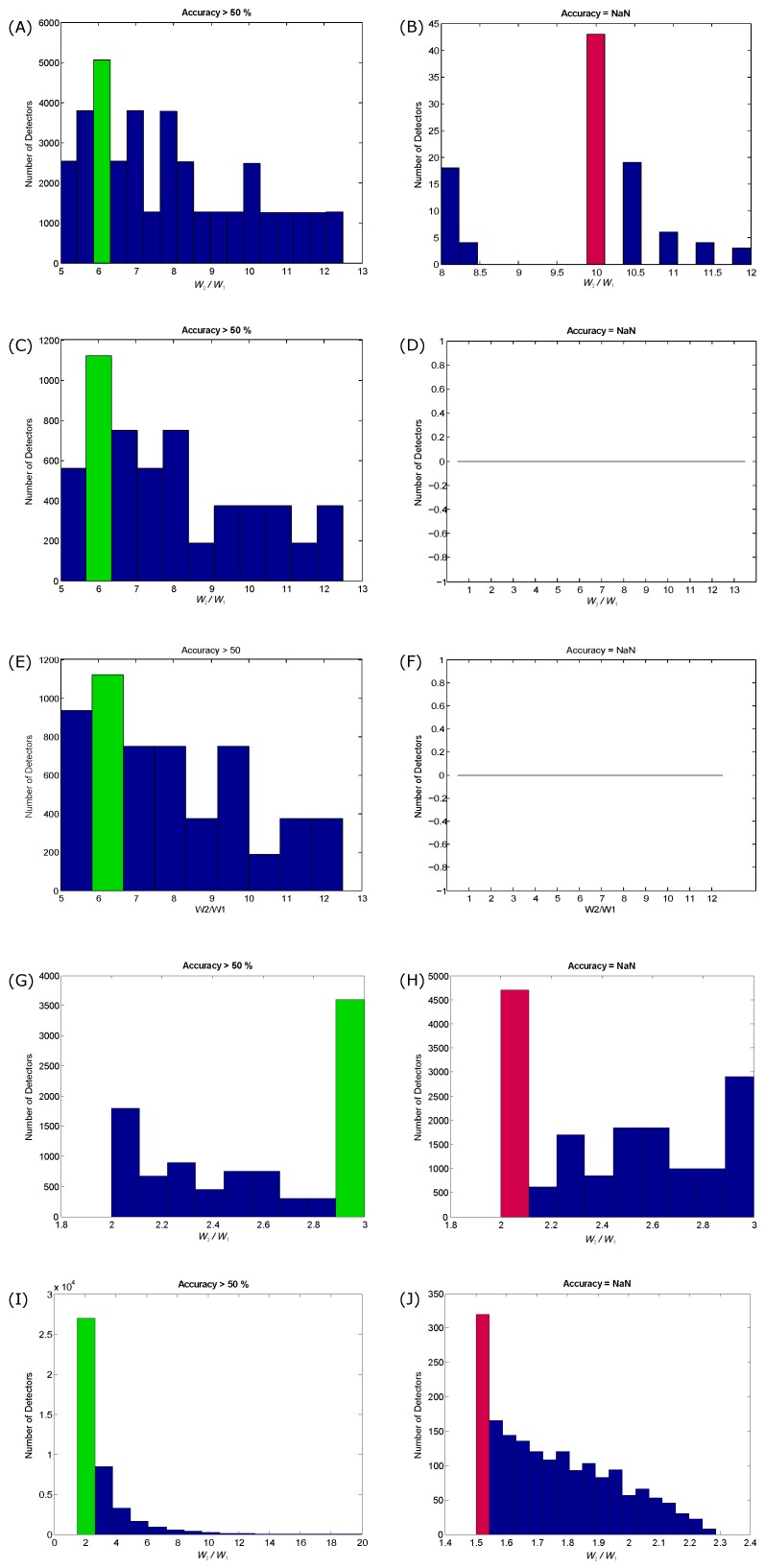
Influence of window sizes on the generation of blocks of interest based on overall accuracy. (**A**,**B**) QRS detection; (**C**,**D**) systolic waves detection; (**E**,**F**) *a* waves detection; (**G**,**H**) c,d, and *e* wave detection; (**I**,**J**) heart sound detection. The left column represents the coupling between the two moving averages by scoring >50% accuracy, while the right column represents non-coupling. The coupling is referred to as accuracy >50%, while non-coupling is referred to as not-a-number (NaN). The green bar represents the most dominant $W_{2}/W_{1}$ ratio in coupling, while the red bar represents the most dominant $W_{2}/W_{1}$ in non-coupling.

In the TERMA method, the use of two moving averages does not always generate blocks of interest. When the two moving averages are able to generate blocks of interest, this is referred to as “coupled” moving averages. To understand and generalize the coupling process between the two moving averages in TERMA, the $W_{2}/W_{1}$ ratio needs to be examined. The coupling between the window sizes of the moving averages over different biomedical signals is investigated, as shown in Figure 5.

To assess the coupling between window sizes, the performance of the created TERMA detectors based on the generated blocks of interest is explored. The performance of TERMA detectors in terms of overall accuracy in detecting a particular event is split into two categories: coupling and non-coupling. The coupling category is when the two moving averages were able to generate blocks of interest and achieved an accuracy >50%, while the non-coupling category is when the two moving averages were unable to generate blocks of interest and achieved an accuracy that is not-a-number (NaN).

Figure 5 demonstrates the effectiveness of the coupling process. For QRS detection in ECG signals, the most dominant ratio that is able to generate blocks of interest is $W_{2}/W_{1}=6$, as shown in Figure 5A. If there is a rough idea about the expected event duration of the QRS complex, the value of $W_{1}$ can be set to be equal to the expected QRS duration and setting the value of $W_{2}$ at six-times that of $W_{1}$. However, if the value of $W_{2}$ is set to be 10-times that of $W_{1}$, there will be no coupling, as shown in Figure 5B, and TERMA fails to detect any event. Interestingly, the same ratio ($W_{2}/W_{1}=6$) is the optimal ratio for detecting the systolic and *a* waves in APG signals, as shown in Figure 5C,E, but TERMA did not fail over the investigated ratios during the training phase, as shown in Figure 5D,F. In the case of detecting c,d, and *e* waves, the optimal coupling ratio is ($W_{2}/W_{1}=3$), as shown in Figure 5G, while the most non-coupling ratio is ($W_{2}/W_{1}=2$), as shown Figure 5H. To detect the heart sounds, the optimal coupling ratio is ($W_{2}/W_{1}=2$), as shown Figure 5I, while the non-coupling ratio is ($W_{2}/W_{1}=1.5$), as shown Figure 5J. These results show that the optimal coupling for TERMA can be achieved using the following inequality:

(7) $$\left(8\times W_{1}\right)\geq W_{2}\geq \left(2\times W_{1}\right)$$

where the lower bound is $\left(2\times W_{1}\right)$ and the higher bound is $\left(8\times W_{1}\right)$.

As can be seen in Figure 5, if the $W_{2}$ is not well defined with respect to $W_{1}$, the detector fails to detect any events. The TERMA testing results, discussed above, are promising for handling the non-stationary effects, low SNR, left bundle branch block, right bundle branch block, premature ventricular contraction, premature atrial and fast heart rate over different biomedical signals. As it is a new concept, there is a need to publish the current results and let the scientific community evaluate its performance on their studies with different types of noise and abnormalities.

### 4.3. Offset β Choice

The offsetting step has been used in the literature as the last stage for most of the event detection algorithms [41,42,43,44,45,46,47]. The performance of the offsetting approach will be affected by low SNR signals [4,48]. Usually, the offset is a fixed value and is experimentally defined [4,41,49,50]. The offsets of these algorithms have been selected based on estimations, which in turn had an impact on the algorithms’ performance. Because the offsetting approach is simple (just an IF-THEN-ELSE statement), researchers used it as a computationally-efficient approach to improve accuracy [4,41,49,50]. The use of a fixed offset to detect a particular event is efficient for stationary biomedical signals with normal beat morphology. Due to severe baseline drifting and the movement of patients, the waveforms of the collected biomedical signal may vary drastically from one heartbeat to the next. Therefore, the probability of missing events is high.

In this work, the offsetting idea is adopted, but implemented differently. The TERMA-based detector uses a signal-dependent offset and not a fixed threshold that is optimized during the training phase. With signal-dependent offsetting (as a percentage of the signal amplitude), the probability of missing events, such as QRS complexes, decreases. It is worth mentioning that in the proposed TERMA method, the offset is applied to the moving average signal and not the original signal, as is usually applied in the literature. The main purpose of the offset in the TERMA method is to reduce the number of generated blocks after applying the two moving averages.

The TERMA offset will slightly shift the output of the second moving average up with the longer window size when applied as a threshold to the first moving average with the smaller window size. The use of a fixed offset to detect particular events, such as QRS complexes, is simple and efficient for stationary ECG signals with normal beat morphology. Due to severe baseline drifting and the movement of patients, an ECG signal waveform may vary drastically from one heartbeat to the next. Therefore, the probability of missing QRS complex is high. With signal-dependent offsetting (as a percentage of the signal amplitude), the probability of missing events, such as QRS complexes, decreases.

**Figure 6:**
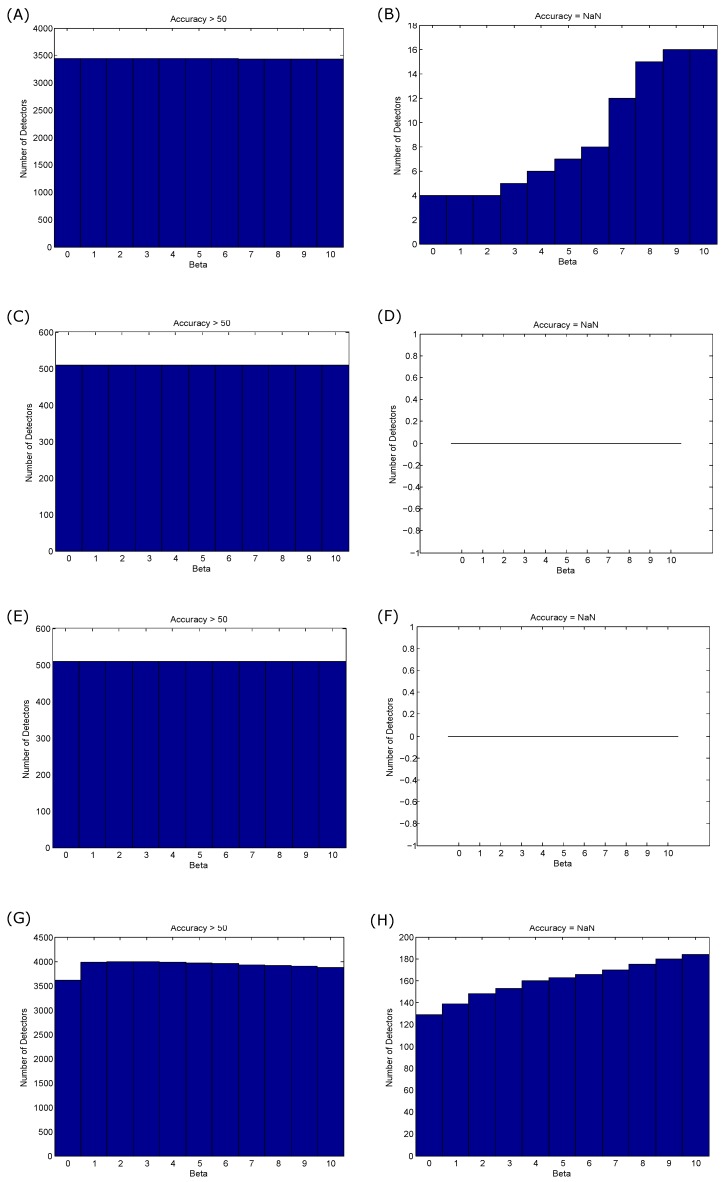
Influence of the offset (*β*) on the generation of blocks of interest on overall accuracy. (**A**,**B**) QRS detection; (**C**,**D**) systolic wave detection; (**E**,**F**) *a* wave detection; and (**G**,**H**) heart sound detection. The impact of the *β* value on the coupling by scoring >50% accuracy is represented in the left column, while the non-coupling is represented in the right column. The coupling is referred to with accuracy >50%, while non-coupling is referred to with not-a-number (NaN).

To assess the impact of the offset on the coupling process, the performance of TERMA detectors is assessed in terms of overall accuracy (>50% and =NaN). Interestingly, the offsetting does not affect the coupling process, as shown Figure 6A,C,E. In other words, the change in *β* does not affect the generation of blocks of interest, but rather improves the overall detection accuracy, especially when the processed signal is relatively noisy. Moreover, the offsetting step in the case of detecting systolic and *a* waves did not cause even one non-coupling case, as shown in Figure 6B,D. In the case of detecting heart sounds, the *β* values had slightly influenced the generation of blocks of interest, as shown in Figure 6G,H.

### 4.4. Battery-Driven Devices

Simplicity is particularly effective when it comes to mobile and battery-driven device computation. Simple analysis methods that achieve high event detection accuracy require less storage and power and are more suitable for battery-driven devices [22,27,51]. It is important to mention that simplicity cannot be achieved unless reliability is also achieved. Simplicity goes hand-in-hand with reliability and must be established in conjunction with simplicity [51].

A simple, yet efficient, event detector is needed to provide a more accurate analysis for wearable devices, point-of-care devices, fitness trackers and smart watches, especially when performance is compared to more complex machine learning solutions [52,53]. Event detection algorithms have been published in the literature [22,27] and compared based on numerical efficiency. It was concluded that the better the numerical efficiency, the faster the algorithm, and vice versa. In other words, the faster the algorithm, the more suitable it is for battery-driven devices.

In the conclusion of [27], the researchers recommended implementing moving averages for batter-driven devices, as they are highly numerically efficient. The implementation of one moving average to detect for simplicity has been discussed in [54,55]. However, the thresholding phase of these one moving average algorithms was complicated and increased the computational complexity [27]. On the other hand the TERMA detector was more efficient and faster than the one moving average algorithms [22].

It is intuitive to think that the use of one moving average is better than using two moving averages, especially for implementation on batter-driven devices. The problem with this approach is the decision making steps required to detect the event. For example, the one moving average-based algorithm utilizes a fixed window size that is determined empirically, and thresholds depend on the accuracy of the heart rate determined in the previous segment [4,22]. However, the TERMA detector does not need to work with a fixed window size; in fact, TERMA processes the whole recording at once. Moreover, TERMA does not need to check the past segments or the previous detection rate [22]. TERMA is advantageous because it uses the second moving average as a threshold to the first moving average, without the need for any complicated thresholding. Therefore, TERMA is promising for a battery-driven device compared to other algorithms.

### 4.5. Optimization Step

Note, the optimization step is time consuming and is not computationally efficient. Perhaps, the calculations of the optimization step can take a place with the use of high-performance computers. However, the implementation of the optimization step is essential and needs to be carried only one time to find the optimal Pareto solution. Once the optimal solution is determined, the TERMA algorithm sets the optimal value as the fixed value for each parameter, and this can be implemented on battery-driven devices with low computation power. Notably, machine learning algorithms usually require high computation for both the optimization step and the algorithm implementation step on battery-driven devices, which can pose a challenge.

The TERMA prior knowledge step is important to practically understand the expected characteristics of the events and noise within the signal. During the optimization, the relationship between the processed signal, added noise, existing events and TERMA parameters is considered. In the test phase, the optimal combination of parameters obtained from the optimization step will be used without any further adjustments. It is important to include a wide variety of waveform and noise to obtain the optimal combination that suits most cases.

### 4.6. Significance of TERMA

We saw how TERMA-based detectors succeeded in detecting events, such as QRS, T, systolic, *a*, *b*, *c*, *d*, *e*, S1 and S2 in different biomedical signals. These biomedical signals were collected using different biosensors with different sampling frequencies in noisy environments. The databases used in the evaluation of TERMA contain signals suffering from: (1) non-stationary effects; (2) low SNR; (3) PACs; (4) PVCs; (5) LBBBs; (6) RBBBs; (7) PAH; (8) heat stress. Based on a review of the current literature, TERMA is the only framework that can be applied to different applications with great success.

The TERMA framework is not only reliable, but also numerically efficient and intuitive. It is easy to track the detection rate and improve accuracy by adjusting five variables. As discussed above the window sizes ($W_{1}$ and $W_{2}$) play a major role in detecting main events in biomedical signals. In other words, setting the values of $W_{1}$ and $W_{2}$ will enable fast analysis of the process. Adjusting the windows sizes provides detailed information of the dominant events in terms of morphology and duration.

Results from this paper lend more insight into implementing the block of interest generation step, by defining the relative values between $W_{1}$ and $W_{2}$ to be $\left[\left(8\times W_{1}\right)\geq W_{2}\geq \left(2\times W_{1}\right)\right]$. These boundaries can be referred to as the “TERMA rate”; it is defining the limits of the lower boundary and higher boundary of successful coupling between two moving averages ($\left[\left(8\times W_{1}\right)\geq W_{2}\geq \left(2\times W_{1}\right)\right]$). This is similar to finding the boundaries for signal sampling (Nyquist rate [$f_{s}$ > 2$f_{max}$]). To clarify the analogy, if we sample a signal at, or above, the Nyquist rate, we can reconstruct the signal. Similarly with the TERMA rate, if the second window size is larger than double the first window size and less than the octuple of the first window size, we can generate blocks of interest and detect main events. Moreover, the TERMA rate can be used to improve the recently-published visualization tool that depends on two moving averages in generating blocks of interest, which is called the eventogram [53].

TERMA framework significance comes from its generic nature for the detection of patterns in any quasi-periodic signal. TERMA framework consists of six independent steps, which can be viewed as LEGO building bricks, and each one of these steps can be modified independently based on the detection problem. Note, the term “LEGO building bricks” is used instead of “LEGO building blocks” because the word “blocks” was used for generating blocks of interest, and it may confuse the reader if “blocks” were used for two different concepts. Thus, TERMA is flexible, universal and can be applied to any periodic or quasi-periodic signals for achieving high accuracy in detecting dominant events within the processed signal. In other words, TERMA is a generic framework that enables researchers to change the filter type, filter order and moving average type based on their application.

Exploring these findings across different types of periodic and quasi-periodic signals that have similar morphologies and characteristics, as in the discussed biomedical signals in this paper, such as the climatic time series [56] (looks like noisy ECG signals), the plant electrical signal [57] (looks like PPG signals), the optical signal [58] (looks like PPG signals), the geophysical signal [59] (looks like the NASDAQ Stock Market signal), the astrophysical signal [60] (looks like noisy ECG signals), the geophysics signal [61] (looks like a noisy heart sounds) and the acoustic and vibration signal [62] (looks like noisy heart sounds), will improve the generalization across the entire signal analysis discipline.

## 5. Conclusions

Event detection in biomedical signals is an important step before analyzing the corresponding waveform in more detail. A new economics-inspired approach for detecting events in biomedical signals is presented. The new algorithm is referred to as TERMA, and its functionality depends mainly on two moving averages similar to those used in economics to examine gross domestic product, employment or other macroeconomic time series. The existence of prior knowledge about the examined waveforms within the biomedical signals will facilitate the adjustment of the window sizes of the two moving averages. Applying the optimization step provides the optimal values of the TERMA, which is recommended for higher detection accuracy. Once the optimal values of TERMA are determined, there is no further tuning needed. Consequently, the validation of the same detector using another dataset without any later parameter tuning can help to obtain more reliable performance results. The performance of the TERMA-based detector is promising. It has been tested on different databases that contain unusual noise and different waveform morphologies. In the literature, it is common to find several algorithms to detect a particular event in a particular biomedical signal. The power of the TERMA-based detector is that it is a generic framework that can be applied to detect different types of events in different biomedical and quasi-periodic signals.
